# Quantitative analysis of the prone stretching and adjusting neck manipulation based on motion capture technology and three-dimensional force measuring tables

**DOI:** 10.3389/fbioe.2025.1610916

**Published:** 2025-10-30

**Authors:** Rui Hong, Xiang-Ming Lin, Chao Yang, Shi-Nian Zhang, Hua Ye, Ying Li

**Affiliations:** ^1^ Gannan Medical University, Ganzhou, Jiangxi, China; ^2^ Department of Human Movement Sciences, Faculty of Physical Education, Gannan Normal University, Ganzhou, China; ^3^ Affiliated Hospital of Nanjing University of Chinese, Nanjing, Jiangsu, China

**Keywords:** prone stretching and adjusting neck manipulation, cervical spondyloticradiculopathy, motioncapture, three-dimensionalforcemeasuringtable, quantification of kinematic parameters

## Abstract

**Objective:**

This study aimed to quantify the kinematic parameters of prone stretching and adjusting neck manipulation using motion capture technology and three-dimensional force measuring tables, thereby providing concrete evidence for standardising and scientifically grounding manual therapy training.

**Methods:**

Ten patients with radiculopathy were recruited, and an experienced physician performed the prone stretching and adjusting neck manipulation. The kinematic data and ground reaction forces of the operator and the patients were synchronized using a Vicon 3D infrared motion capture system and a Kistler three-dimensional force measuring table.

**Results:**

The results of this study showed that when performing the “prone stretching and adjusting neck manipulation” manoeuvre, the maximum force loaded vertically by the active hand in the left and right rotational pulling (wrenching phase) was 551.5 N, and the minimum was 418.3 N, with a mean value of (476.75 ± 33.11) N. The maximum force loaded vertically in the left and right rotational pulling was 400.43 N, and the minimum was 182.4 N. The mean value was (274.79 ± 52.08) N. The manipulation trigger time was (0.35 ± 0.03) s, the maximum rotation angle was (73.7 ± 1.34)°, and the subject’s neck extension was (4.39 ± 1.02) mm.

**Conclusion:**

The “prone stretching and adjusting neck manipulation” is time-sensitive, short. The manoeuvre was divided into three phases: the stretching phase, the triggering phase, and the return phase. It involves both the active hand and the auxiliary hand, and the maximum force loaded in the vertical direction of the active hand is consistent in different operating directions.

## Introduction

Cervical spondylotic radiculopathy is one of the more common types of cervical spondylosis, accounting for approximately 60%–70% of all cervical spine disorders (Group, 2015). It is usually caused by herniation of cervical intervertebral discs and degenerative changes in the intervertebral joints, which lead to compression or irritation of the nerve roots in the corresponding segments, thus triggering a series of symptoms and signs. In recent years, the incidence of cervical spondylotic radiculopathy has shown a trend of gradual increase (Li et al., 2024; Luyao et al., 2022; Pu et al., 2023; Zhang et al., 2023), seriously affecting 0.35% of the global population (Salemi et al., 1996). The pathogenesis of cervical spondylotic radiculopathy has not been fully explored by modern medicine, and it is currently believed to be mainly related to mechanical compression, chemical radiculitis and autoimmune regulation (Petersen et al., 2017). Traditional Chinese medicine theory, on the other hand, attributes the causes of this disease to two categories of internal and external causes. At this stage, the treatment of cervical spondylotic radiculopathy is mainly based on conservative treatment, such as joint release, acupuncture therapy (Peng et al., 2023), massage therapy (Yu et al., 2024), and spinal traction (Chen et al., 2021). These manipulative treatments can have significant clinical therapeutic effects on cervical spondylotic radiculopathy and have earned wide acceptance among patients due to their clear efficacy, affordable treatment cost, and mild side effects (Kuligowski et al., 2021).

The “prone stretching and adjusting neck manipulation”, as one of the traditional massage techniques, originated from the “four-finger massage” academic school of massage (Yan and Zhang, 2020), which combines the traditional characteristics of traditional Chinese medicine, and integrates the modern cervical spine anatomy and its biomechanical characteristics, combined with the use of clinical practice, and is applied to stretch and readjust the cervical spine in the prone position. This manoeuvre has been used in clinical practice for many years (Feng et al., 2018; Ren, 2021; Xiong et al., 2017) and has significant therapeutic effects. This technique employs specific traction and rotational movements to produce beneficial biomechanical effects on cervical vertebral structures. Research indicates (Savva et al., 2021; Yufeng et al., 2022) that such manipulations alleviate symptoms of cervical spondylotic radiculopathy by enlarging the intervertebral foramen area and reducing nerve root compression. Specifically, during the traction phase, sustained pulling increases intervertebral disc height and lowers intradiscal pressure; while the manipulation phase employs high-velocity, low-amplitude thrusts to generate transient cervical rotation and lateral flexion, further widening the neural foramen and reducing mechanical compression on the nerve roots (Moustafa et al., 2022). Additionally, this technique modulates local soft tissue tension, improves blood circulation, and alleviates inflammation and oedema surrounding the nerve roots, thereby decreasing nerve root tension (Jiang et al., 2012). These combined mechanisms contribute to restoring the normal physiological environment of the nerve roots, relieving pain and radicular symptoms. Previous studies lacked quality control of the manipulation, the manipulation standards were not quantified, and there was a lack of rigorous randomized controlled trials and efficacy evaluations, which is not conducive to the further promotion and application of the manipulation. In this study, we aimed to quantify the kinematic parameters of the prone stretching and adjusting neck manipulation using motion capture technique and 3D force measuring tables, to investigate its effect on the biomechanics of the cervical spine, and to provide a scientific basis for the clinical application of this method.

## Materials and methods

### Vicon 3D infrared motion capture system and 3D force measuring table

**FIGURE 1:**
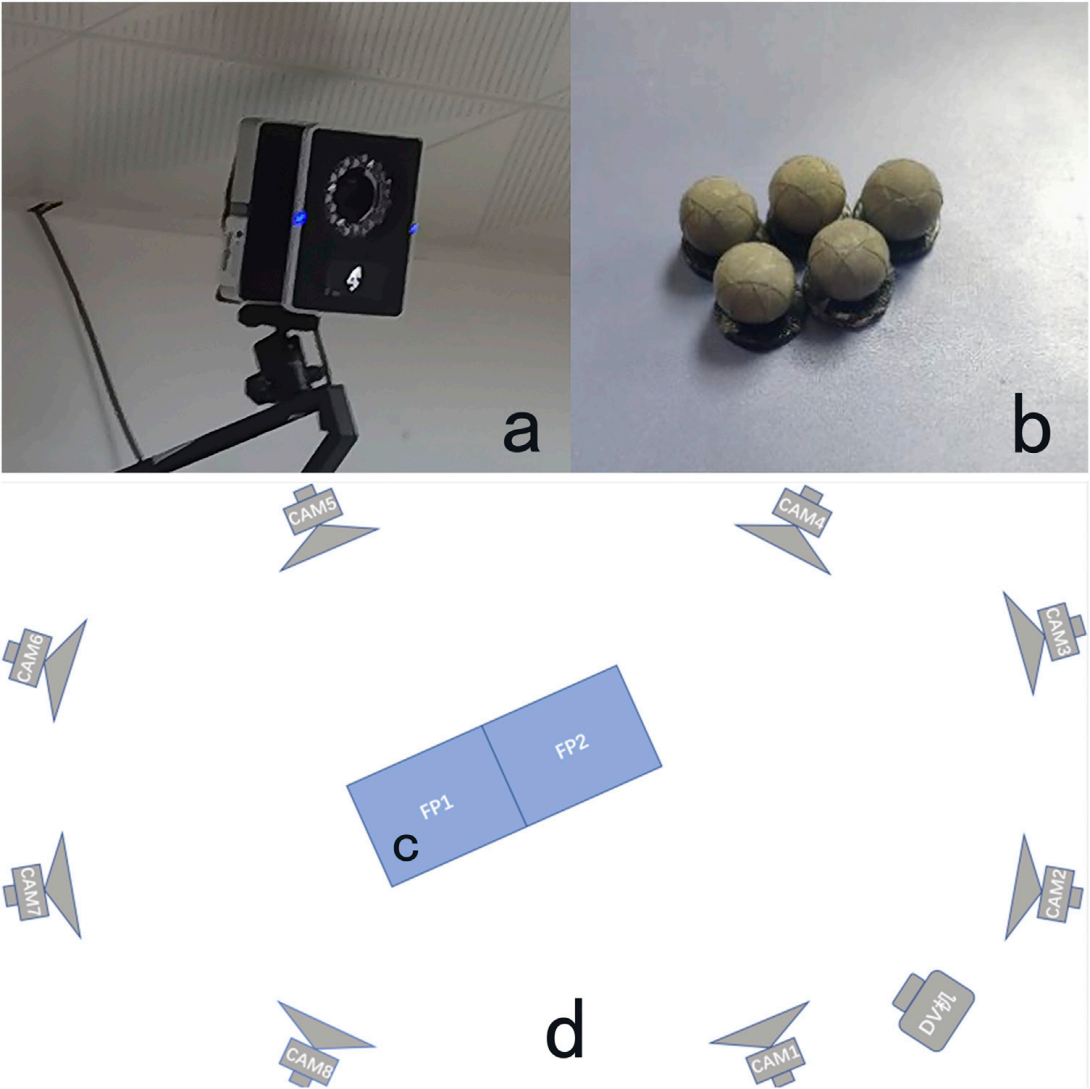
Vicon infrared camera **(a)**, Marker point **(b)**, 3D force measuring table **(c)** and Schematic diagram of indoor venue setting **(d)**.

The Vicon 3D IR Motion Capture System consists of eight high-resolution infrared cameras, a DV sync camera (Figure 1a), 12 mm diameter reflective Marker balls (Figure 1b), and a host PC with Vicon Nexus software. A Vicon Nexus was utilized for kinematic data acquisition in the experiments, with the sampling frequency set at 200 Hz, and the signals were processed using the relevant analysis software. The 3D infrared motion capture system used in this test relies on the capture of reflected light from the Marker points pasted on the subject’s body. Considering its high requirement for the stability of light in the external environment, this study chose an indoor laboratory with relatively stable light as the research site, in Room 703 of the Guangdong Provincial Engineering Research Center for Auxiliary Aids for Sports at the Guangzhou Institute of Physical Education. Indoor site setup: 8 Vicon infrared cameras, one DV Sync Capture Unit and two 3D Force measuring tables set as shown in Figure 1d. A Kistler three-dimensional force measuring table (Figure 1c), manufactured by a German company, was also used in this experiment to determine the time-dependent changes of the ground reaction forces in the anterior-posterior, left-right, and vertical directions during the application of force to the subject by the “prone stretching and adjusting neck manipulation” technique. Two Kistler 3D force measuring tables, each measuring 60 cm × 40 cm and with a data acquisition frequency of 1,000 Hz, were embedded in the floor side-by-side to synchronize with the Vicon infrared capture system to capture the ground reaction forces and connect to a PC to facilitate the subsequent analysis of kinematics-related parameters (He, 2023).

### Motion analysis software

Using the Visual 3D v6 x 64 version, it is able to process and calculate the kinematic parameters collected by Vicon and the ground reaction force data collected by the Kistler 3D force platform using inverse dynamics and generate smooth graphs to obtain the required biomechanical parameters.

## Methods

### Selection of caster

1 clinician with at least 10 years of clinical experience and proficiency in the “prone stretching and adjusting neck manipulation” manoeuvre.

### Selection of subjects

In the outpatient department of Guangzhou Sports Institute, 10 patients with cervical spondylotic radiculopathy who met the study criteria were recruited. The inclusion criteria were by the diagnostic criteria for cervical spondylotic radiculopathy in Chinese and Western medicine; the age was between 18 and 60 years old, and the gender was unlimited; they did not receive massage and other related treatments for 7 days before starting the treatment. The subjects voluntarily fulfilled the commitment procedures and signed the consent form on the basis of their informed consent. Before the experimental operation, the experimental steps were explained to the subjects, and the precautions during the experimental process were explained so that the data could be collected accurately.

## Experimental procedures

### Acquisition instrument commissioning and warm-up

Before conducting the experiment, the camera, DV camera and force table of the Vicon 3D infrared motion capture system need to be warmed up. At the same time, it is necessary to organize the experimental site to reduce the interference of stray points, and then complete the spatial position calibration of the camera and the synchronization of the acquisition instrument.

Site calibration: A T-shaped calibrator was used, which was continuously waved in the field for dynamic calibration, and horizontally placed and stuck in a corner of the force measuring table for static calibration. Calibration of the instrument and the calibration site is performed in space, in the horizontal plane, and at the origin of the coordinates. The X-axis represents the frontal axis, the Y-axis represents the sagittal axis, and the Z-axis represents the vertical axis in the three-dimensional space.

### Paste marker reflective balls

**FIGURE 2:**
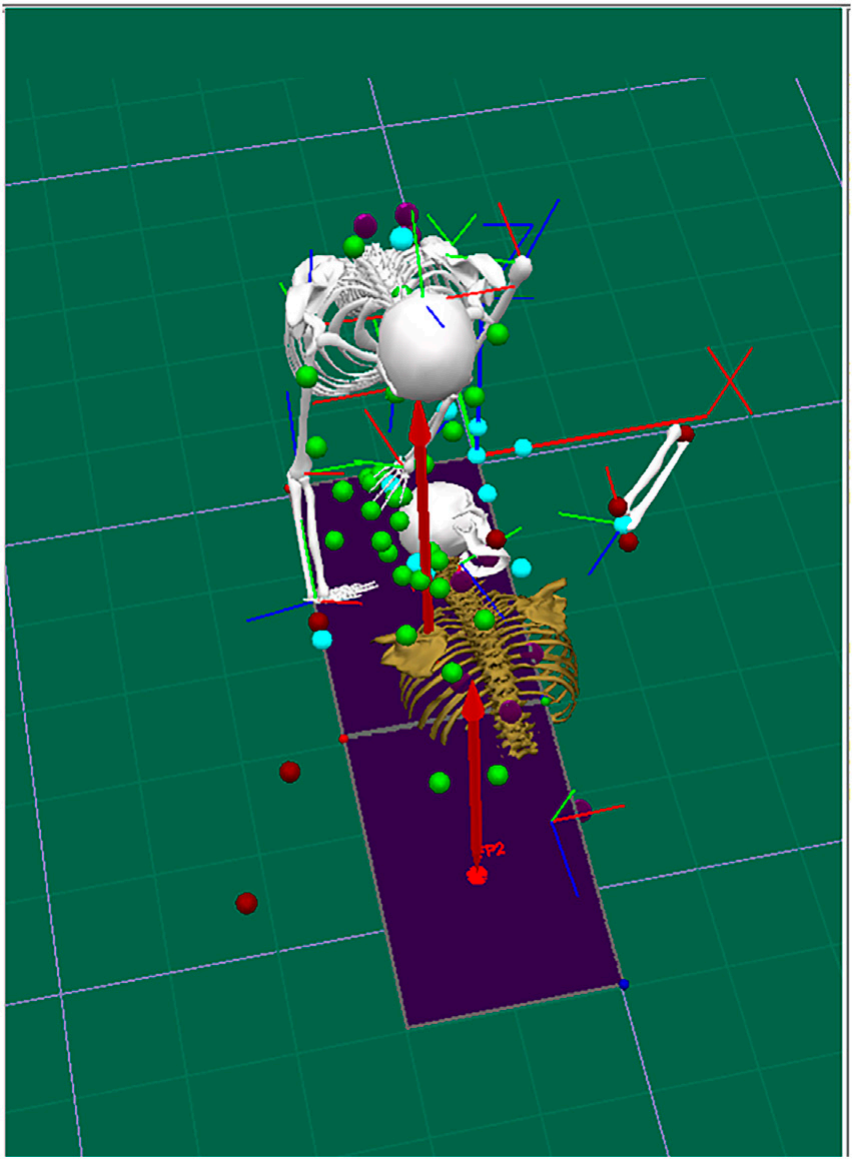
Schematic diagram of a three-dimensional model for manual manipulation.

In this experiment, we based on the whole-body skeleton model that comes with the Vicon motion capture system and modified it with the Visual 3D data import and experimental purpose, and finally determined the standard of marker placement that applies to the needs of this experiment. The reflective markers were basically placed symmetrically, with 20 points for the subject and 24 points for the applicator, and the specific positions are shown in Tables 1, 2. To ensure the uniformity of the marker points, the same operator was responsible for pasting the markers on all subjects and applicators. The specific operational details of the treatment process can be seen in Figure 2.

**TABLE 1:** Subject marker reflective ball paste position.

	Specific location	Quantity (pcs)
Head	Crown of the head	1
	Temporal	Left and right 1 each
	Occipital bulge	1
	Retroauricular mastoid process	Left and right 1 each
Neck	Fifth cervical vertebra under the spinous process	1
	Shoulder peak	Left and right 1 each
	Subscapularis (anatomy)	Left and right 1 each
Trunk	Tenth thoracic subacromial vertebra	1
Upper limb	Medial epicondyle of the humerus	Left and right 1 each
	Epicondyle of the humerus	Left and right 1 each
	Midpoint of the line between the radial tuberosity and the ulnar tuberosity	Left and right 1 each
Pelvic	Posterior superior iliac spine (anatomy)	Left and right 1 each
Total		20

**TABLE 2:** The practitioner makes a reflective ball paste position.

	Specific location	Quantity (pcs)
Neck	Seventh cervical vertebra, subspinous	1
Trunk	Sternal shank	1
	Shoulder peak	Left and right 1 each
	Subscapularis (anatomy)	Left and right 1 each
Upper limb	Medial epicondyle of the humerus	Left and right 1 each
	Epicondyle of the humerus	Left and right 1 each
	Stem of the radius (anatomy)	Left and right 1 each
	Ulnar tuberosity (anatomy)	Left and right 1 each
	Third metacarpophalangeal joint	Left and right 1 each
	Projection of the heel of the palm on the back of the palm	Left and right 2 each
	Upper arm tracking point, forearm tracking point	Left and right 2 each
Total		24

All tracking points are only meant to assist in tracking rigid bodies and do not have a specific location.

### Modelling

**FIGURE 3:**
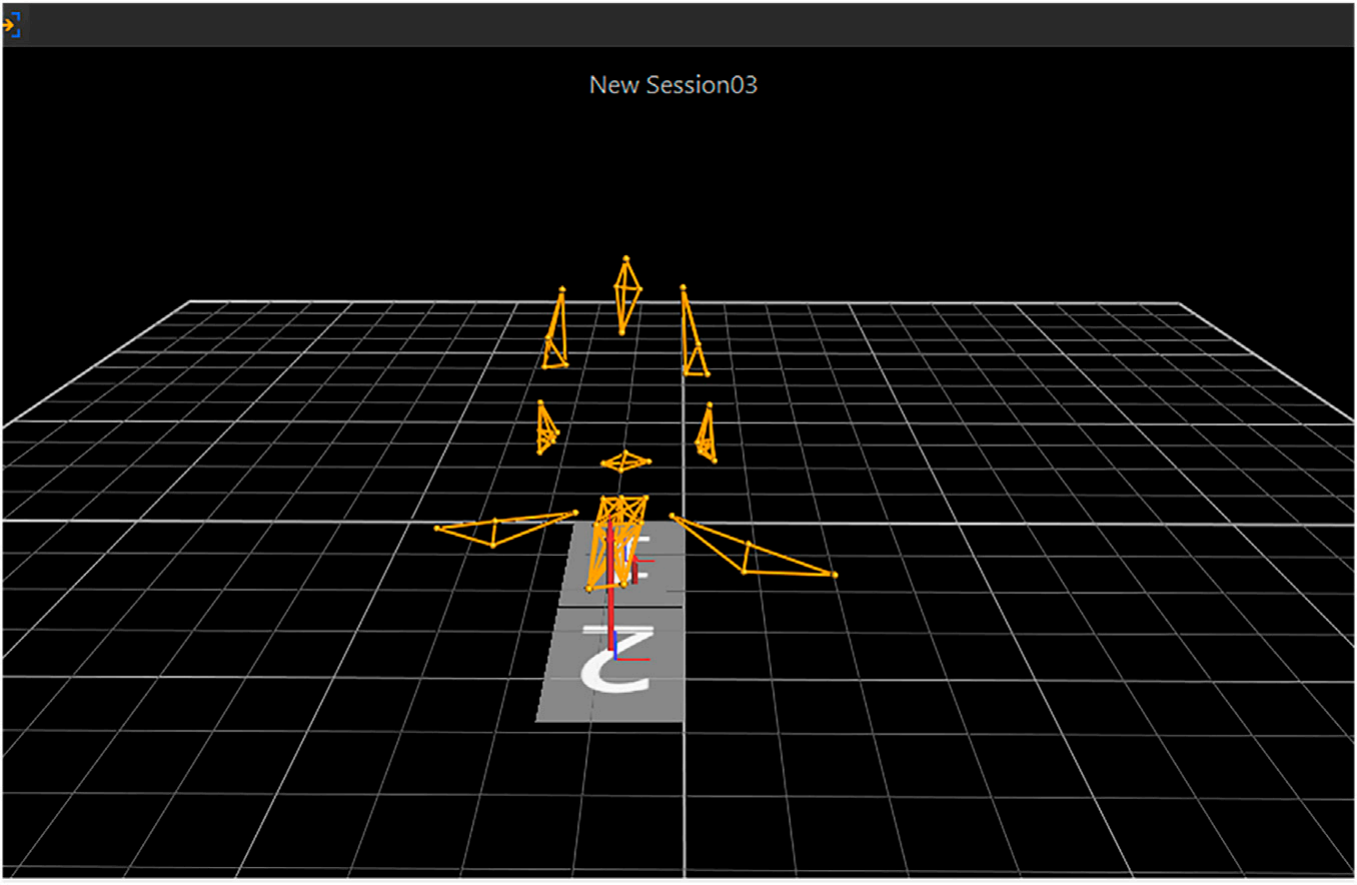
Modeling of the upper body of the practitioner and the subject.

Using the Vicon Nexus software, according to the characteristics of the “prone stretching and adjusting neck manipulation”, both the operator and the subject were modelled with the upper body, as shown in Figure 3.

### Prone stretching and adjusting neck manipulation manoeuvre

The subject is placed in a prone position, with the head on ergometer 1, a soft pillow under the chin, and the chest on ergometer 2. The head should be rotated to the left to maximize the angle of rotation while keeping the left upper extremity extended and the right upper extremity naturally placed at the side of the body, and the whole body relaxed as much as possible. The operator adopts a kneeling position, pushes the subject’s occipitotemporal region with the root of the palm of the right hand, and the root of the palm of the other hand is pressed against the subject’s C7-T3 spinous processes; the subject is instructed to give the neck a neck retraction during inhalation, and during exhalation, the operator reverses the downward and outward application of a quick, light, and controlled inch force with both hands. Then the subject’s head is turned to the right side and operated in the same way (Ren, 2021).

### Experimental observation indicators

The Vicon 3D infrared motion capture system is used to capture the marker points on the human body surface in real-time, and the captured information is processed by the video controller, and the results are entered into the 3D data capture workstation. The 3D force measuring table is utilized to capture the reaction force on the ground during the manipulation in real-time. The kinematic data were obtained by Visual 3D software, and the obtained data were plotted to obtain the parameters of important phases and analyzed as follows: the time-force curve of the manipulation process of “prone stretching and adjusting neck manipulation”, the division of the time phase of the manipulation operation, the peak value of the loading force of the manipulation, the triggering time, the angle of rotation of the trigger, and the magnitude of the subject’s neck extension. Three axial forces were generated in the 3D force measuring table: X-axis represented the force in the left and right directions of the horizontal plane, with the left being negative and the right positive; Y-axis represented the force in the front and back directions of the horizontal plane, with the front being positive and the back negative; and Z-axis represented the force in the vertical direction, with the upper being positive and the lower being negative.

### Statistical methodology used

The SPSS 26.0 statistical software package was used for data processing in this experiment. Measurement data were tested for normality using the Shapiro-Wilk test and for chi-square using the Levene test. Mean ± standard deviation was used when conforming to normal distribution and chi-square, paired T-test was used for pre-and post-operation comparisons, and corrected T-test was used for chi-square data. P < 0.05 was considered a statistically significant difference, and the significance level of the test was 0.05 (bilateral).

## Result

### General information on subjects

General information on subjects can be obtained in Table 3.

**TABLE 3:** General information of subjects.

Number of examples	Gender (cases)		Age	Disease duration
	Male	Female	(Year, $\bar{x}\pm S$ )	(Week, $\bar{x}\pm S$ )
10	5	5	40.9 ± 8.28	28.9 ± 12.9

### Time force variation curve of the manipulation process of force measuring platforms 1 and 2

**FIGURE 4:**
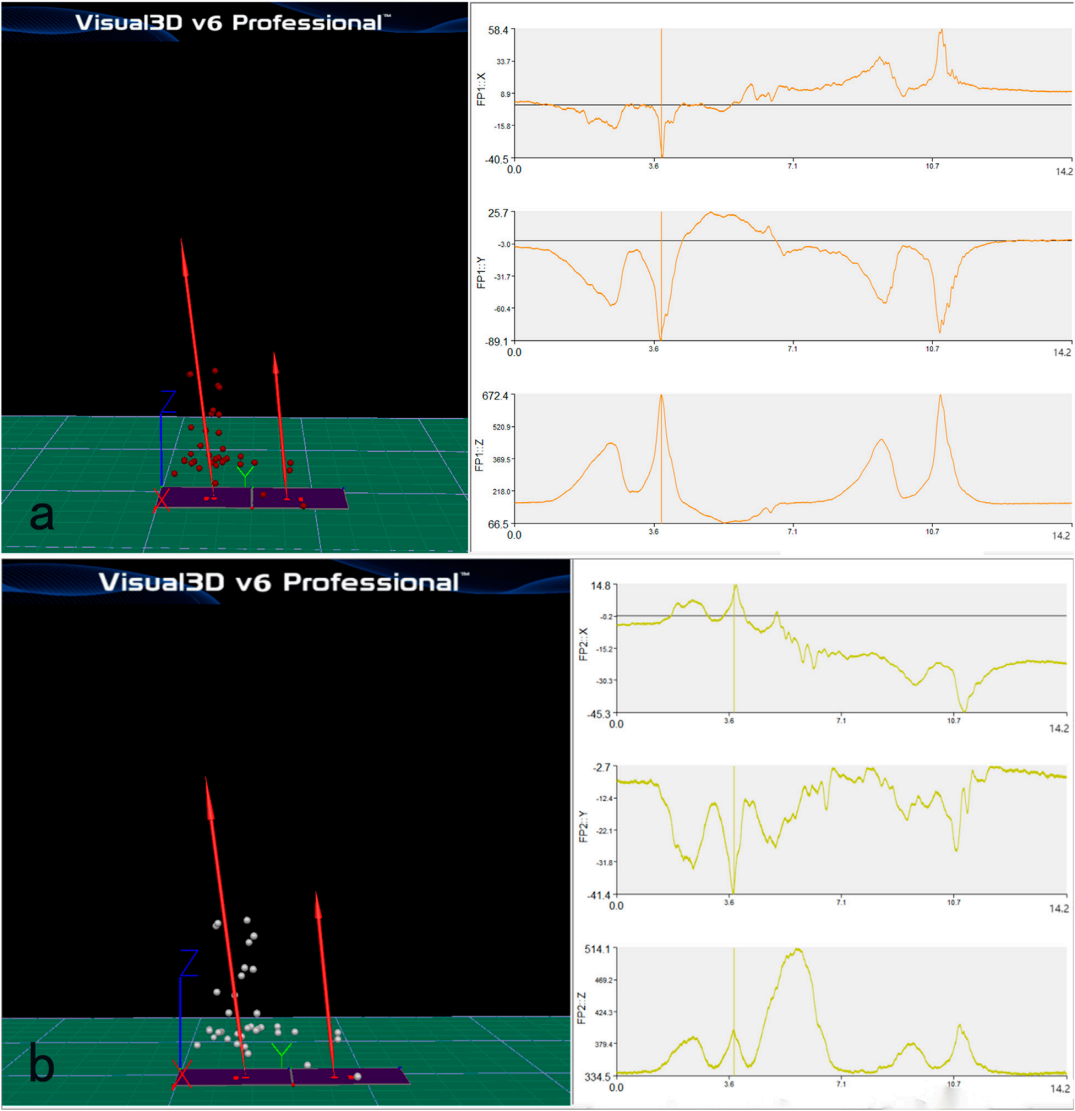
Time force variation curve of the manipulation process of force measuring platform 1 **(a)** and 2 **(b)**.

In the “prone stretching and adjusting neck manipulation”, the hand that presses the occipitotemporal region is the main hand, which is the main force-generating hand, and the hand that presses the thoracic vertebrae is the auxiliary hand, which is mainly used to fix the thoracic vertebrae. By analyzing the data of the two force measuring stations (Figure 4), the amplitude of the change of the combined force of the force measuring station 1 (the active hand) is obviously larger than that of the force measuring station 2 (the auxiliary hand), so we mainly focus on the value of the force measuring platform 1 in our analysis.

When the practitioner operates the “prone stretching and adjusting neck manipulation”, it will generate three axial forces in the X, Y and Z axes, the X and Y axes represent the ground reaction force in the horizontal plane in the anterior-posterior and left-right directions, and the Z axis direction is the ground reaction force in the vertical direction. According to Newton’s third law, the action and reaction forces are always equal in size and opposite in direction, so the magnitude of the ground reaction force in the Z-axis direction can reflect the magnitude of the pressure in the vertical downward direction of the technique. After analyzing the data of 10 subjects, the vector sum of the X and Y axes is smaller than the absolute value of the vertical force, therefore, the following experiments are based on the Z-axis direction, the vertical ground force (which can reflect the pressing force) as the main body of the kinematic analysis.

### Manipulation of the time-phase division

**FIGURE 5:**
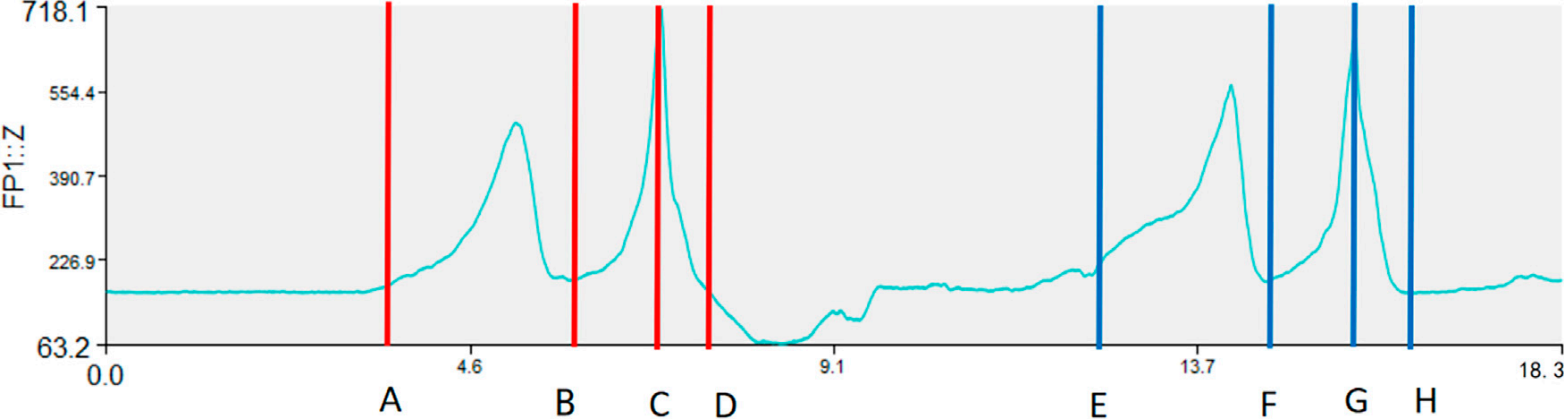
Vertical Ground Reaction Force and Time Distribution Diagram.

Figure 5 shows the change curve of the vertical force during the operation of the “prone stretching and adjusting neck manipulation”, the horizontal coordinate is the time axis (unit: second), the vertical coordinate is the vertical force (unit: Newton), and the light blue line is the trend of the vertical ground reaction force over time, which can reflect the trend of the vertical pressure of the active hand.

Take the left-handed pulling trigger as an example, and divide the important nodes in the process of manipulation into four points, A, B, C, and D. The right-handed trigger can refer to the left-handed pulling trigger, and points E, F, G, and H correspond to points A, B, C, and D, respectively. Point A: the point at which the practitioner starts stretching the subject’s neck. It is the initial position corresponding to the manoeuvre when the subject and the practitioner are in a ready position. Point B: The turning point from the end of the stretch to the instantaneous force generation. Point C: The turning point from the peak force value during the applicator’s force generation to the point at which the ground reaction force begins to diminish, this point may reflect the peak value by pressure (vertical direction). Point D: The endpoint of the prone stretching and neck adjustment manoeuvre, where the applicator’s force-generating hand is returned to position and the force is withdrawn, shown as the initial force value. According to the four key turning points mentioned above, the vertical ground reaction force curve of the whole prone stretching and adjusting neck manipulation process can be divided into three parts: A-B stretching phase, B-C triggering phase, and C-D return phase.

### Comparison of the maximum force loaded in the vertical direction of the active hand for left and right hand pulling (triggering phase and stretching phase)

Ten different subjects were tested, and the obtained data were plotted separately by Visual 3D software. According to the time-force graph, the maximum force values of the vertical direction of the applicator’s left- and right-rotation pulling trigger hand were obtained, and the maximum force value of the loaded force = maximum force peak value - initial force value. The initial force value is mainly related to the weight of the subject’s head and neck, and there are individual differences between different subjects.

In a comparison of the maximum force values loaded in the vertical direction by the active hand for the left and right rotational draft (triggering phase and stretching phase) (Table 4), the difference was not statistically significant using the paired samples T-test (P > 0.05), indicating that the maximum force values loaded vertically by the manoeuvre on the subject during triggering/stretching are consistent between the left and right rotational drafts. In this experiment, the maximum force value of the prone stretching and adjusting neck manipulation triggering phase loaded in the vertical direction was 551.5 N, the minimum value was 418.3 N, and the mean value was (476.75 ± 33.11) N. The maximum force value of this method drafting phase loaded in the vertical direction was 400.43 N, the minimum value was 182.4 N, and the mean value was (274.79 ± 52.08) N.

**TABLE 4:** Maximum force values for vertical loading of left and right rotation.

	Direction	Sample volume	Load maximum force value	t-value	P-value
Triggering phase	Levitation	10	481.14 ± 42.49	0.735	0.481
	Right rotation	10	472.36 ± 21.62		
Stretching phase	Levitation	10	268.01 ± 46.97	−1.024	0.333
	Right rotation	10	281.58 ± 58.46		

### Performer’s trigger time for “prone stretching and adjusting neck manipulation”

The time of the “rapid inch force” during the “prone stretching and adjusting neck manipulation” is calculated by analyzing the change in the vertical displacement of the Marker point of the right shoulder peak with the active hand of the right hand of the practitioner in the case of left-rotation pulling and triggering.

**FIGURE 6:**
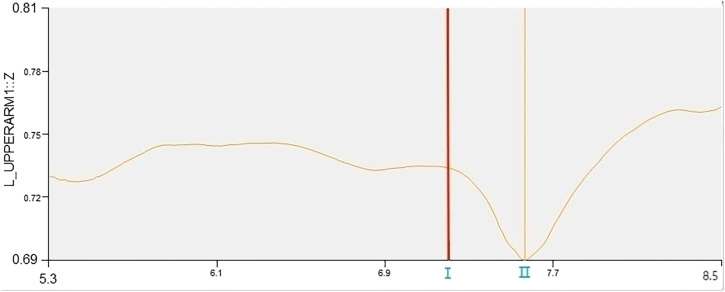
Right shoulder peak displacement curve.

As shown in Figure 6, Ⅰ is the position of the Marker point of the right shoulder peak at the start of the force at the moment of triggering, and Ⅱ is the peak displacement of the Marker point of the right shoulder peak in the vertical direction during the triggering manoeuvre. The time from Ⅰ to Ⅱ was taken as the trigger time of “prone stretching and adjusting neck manipulation”, and the trigger time was 0.35 ± 0.03s, and the statistics are shown in Table 5.

**TABLE 5:** Manual switching time and neck extension amplitude of subjects.

Enterprise	Sample volume	Result
Trigger time	10	0.35 ± 0.03
Drafting amplitude	10	4.39 ± 1.02

### The subject’s neck extension amplitude

**FIGURE 7:**
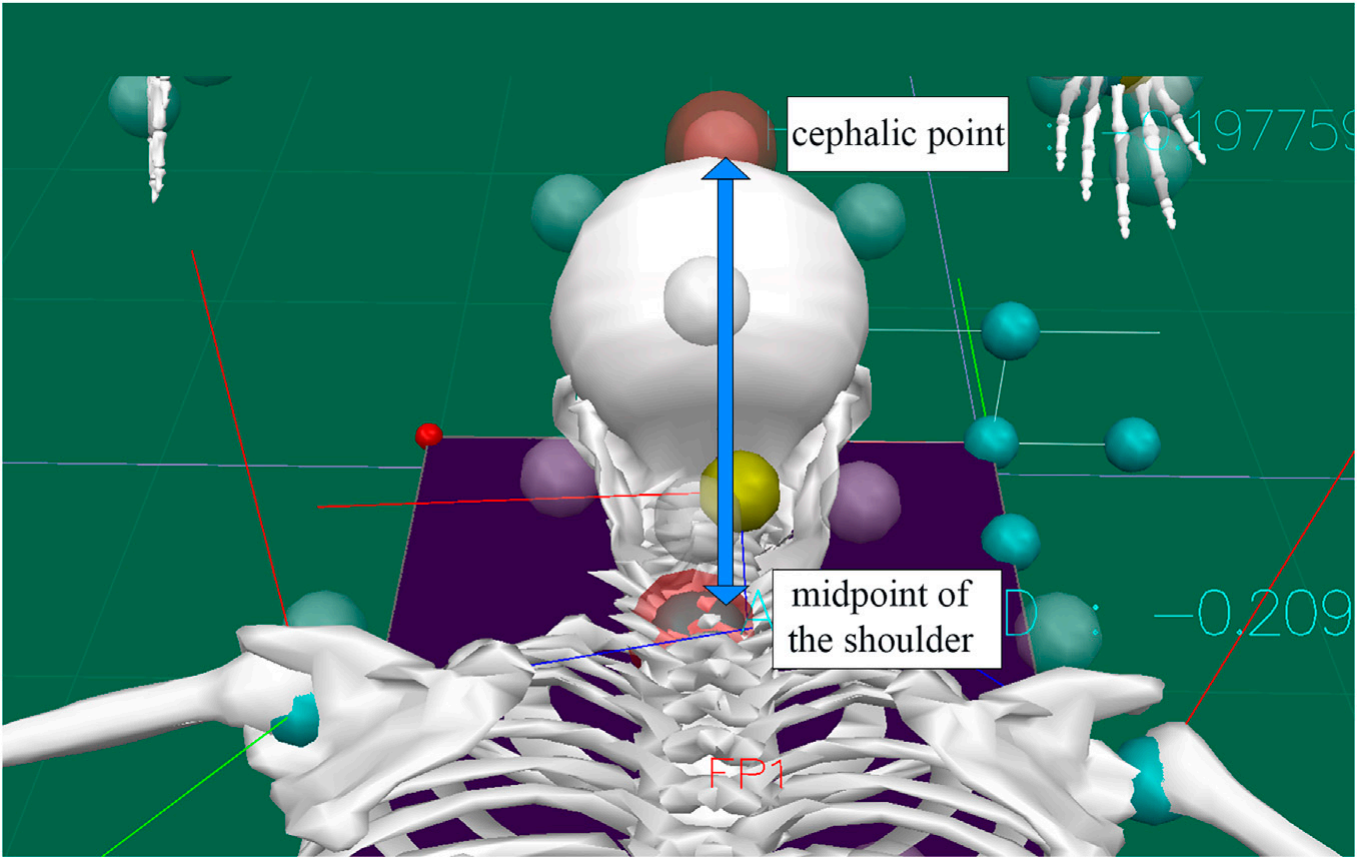
Cephalic point and midshoulder point.

As shown in Figure 7, the Marker head vertex and mid-shoulder point are constructed by Visual 3D motion analysis software through the mid-point of the line connecting the left and right shoulder peak Marker points, which are roughly located at T1 in the human body. In the initial position, the distance from the top of the head to the mid-shoulder point is used as the base value before the “prone stretching and adjusting neck manipulation”, and the maximum stretching amplitude is the maximum distance between the two points. Manipulation on the neck retraction amplitude, that is, the difference between the two, the left side of the manipulation, on the 10 subjects retraction amplitude, the retraction amplitude of 4.39 ± 1.02 mm, statistics are shown in Table 5.

### The subject’s neck rotation angle

**FIGURE 8:**
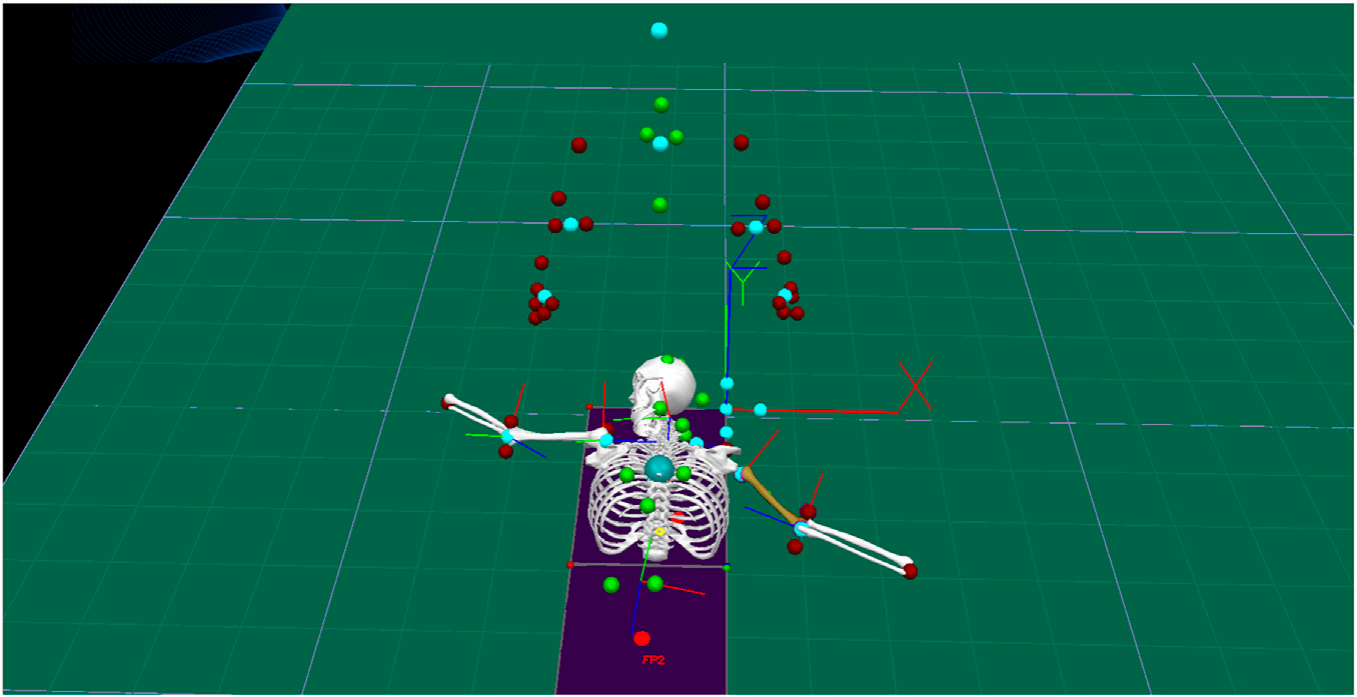
Rigid head and rigid chest.

The Marker points of the head (top of the head, temporal region, external occipital bump, and postauricular mastoid) were used as the reference to determine the head’s rigid body, and the Marker points of the chest (acromion, subscapular angle, and infraspinous process of the 10th thoracic vertebrae) were used as the reference to determine the chest rigid body (Figure 8), and with the help of the Visual 3D motion analysis software, the rotation angle of the head with respect to the chest was calculated to analyze the subject’s before and after manipulation. The maximum left rotation angle of the subject before and after the maneuver was analyzed, 68.6° ± 2.37° before the maneuver and 73.7° ± 1.34° after the maneuver (Table 6).

**TABLE 6:** Comparison of cervical rotation angles before and after manual manipulation.

Enterprise	Sample volume	Maximum left rotation angle	t-value	*P*-value
Pre-operational	10	68.6 ± 2.37^#^	−11.769	0.000
Post-operational	10	73.7 ± 1.34		

Comparison with post-operation ^#^P < 0.05.

A comparison of the maximum left rotation angle of the cervical spine before and after manipulation of the “prone stretching and adjusting neck manipulation” was performed using the paired-sample T-test, and the results showed that the P-value was 0, which was less than 0.05, so the difference was statistically significant (P < 0.05), which indicated that the manipulation process could increase the maximum angle of the left rotation of the subject.

## Discussion

The quantitative study of massage manipulation can lead to standardization and normalization of massage manipulation, which not only contributes to the inheritance of manipulation and the transformation of the results of manipulation (Liang et al., 2020) but also provides a new perspective for the massage treatment of cervical spondylosis radiculopathy. The quantification of mechanical parameters of massage manipulation is the focus of quantitative research, and many scholars have conducted a series of quantitative studies based on the mechanical characteristics of massage manipulation (Gao et al., 2018; Huang et al., 2021; Li et al., 2017; Lu et al., 2022). The force generated during the actual operation of massage manipulation is a three-dimensional force, but previous studies such as the multi-point film pressure test system usually measure a one-dimensional force perpendicular to the contact surface, compared to a three-dimensional force measuring table that is more comprehensive and can better measure the force in a three-dimensional direction. The three-dimensional force table demonstrated its advantages in related studies (Owens et al., 2017; Y, 2014) and the three-dimensional force is also used in this experiment for quantitative analysis of relevant motion data. In this experimental study, three-dimensional force measuring tables were used to collect the dynamic changes of mechanics during the operation of the “prone stretching and adjusting neck manipulation”, and the frequency of collection was 1,000 Hz. According to the operation characteristics of the technique and the time-force change curve, the technique was divided into three parts: the stretching phase, the triggering phase, and the return phase, and the preliminary quantification of the mechanical parameters of the technique was carried out.

Motion capture technology is a kind of technology used to accurately measure the movement condition of moving objects in three-dimensional space (Liu et al., 2022), which has the characteristics of high accuracy and can realize dynamic three-dimensional motion analysis (Lu et al., 2023; Reis and Lochmann, 2015). At present, motion capture technology has been gradually applied to the study of massage manipulation, using which the strength, angle, displacement, and time of the operation of massage manipulation can be visualized, so that the study of massage manipulation can be standardized, data-driven, and visualized, and get rid of the empirical-dominated research mode (Gao et al., 2020). Some studies (Chatzitofis et al., 2019; Gao et al., 2018; Li et al., 2019; Lin et al., 2023; Yan et al., 2020) have used motion capture technology and demonstrated its significant advantages, and the 3D motion capture system applied in this study adopts dynamic linear technology with an acquisition frequency of 200 Hz, which ensures the accuracy of the calibration and the precision of the trajectory tracing and is capable of carrying out detailed digitized displays and animation formation. The experiment was based on the motion capture technology for the comparison of left and right side contrast manoeuvres, and the number of Marker points selected was large and standardized, and the test object was not only the subject but also the movement of the operator during the treatment process was recorded so that the measurement results were true and accurate. Motion capture technique also has better reliability and validity than traditional measurement technique measurements (Feng et al., 2019), such as the measurement of cervical spine mobility (Inokuchi et al., 2015). In this experiment, the cervical spine rotational mobility was measured using the motion capture technique, and the cervical spine mobility calculated by the software is the true value of the relative angle between the cervical spine and the torso after modelling and coordinate system establishment (Feng et al., 2018). In the “prone stretching and adjusting neck manipulation”, the manipulation trigger time is determined by the displacement and speed change of the active hand, which will be more accurate. In the experimental process, in order to prevent the loss of Marker points, attention needs to be paid to the adjustment of the position of the Marker points, so the Marker points of the acromion were used for the measurement.

The results of this study showed that when performing the “prone stretching and adjusting neck manipulation” manoeuvre, the maximum force loaded vertically by the active hand in the left and right rotational pulling (wrenching phase) was 551.5 N, and the minimum was 418.3 N, with a mean value of (476.75 ± 33.11) N. The maximum force loaded vertically in the left and right rotational pulling was 400.43 N, and the minimum was 182.4 N. The mean value was (274.79 ± 52.08) N. This may indeed present potential risks and hazards; however, the force applied during this study constitutes a torsional force acting upon the entire head-neck and thoracic-cervical region, rather than directly impacting the vertebral bodies. It is distributed across the whole of the cervical spine, functioning as a torsional load. Based on our prior finite element modelling research, we found that following simulated manual loading, the actual stresses applied to each segment of the cervical spine remained within safe threshold limits, thereby confirming the safety of this manipulation technique.

In this experiment, the time-force graph of the “prone stretching and adjusting neck manipulation” manoeuvre, the time phase of the manoeuvre, the peak of the loading force of the manoeuvre, the trigger time, the angle of the trigger, and the magnitude of the subject’s neck extension were analyzed to explore the quantitative study of the “prone stretching and adjusting neck manipulation” manoeuvre. A total of 20 time-force curves of the manipulation process were obtained in this experiment, and 10 graphs were obtained from each of the force measuring Tables 1, 2. According to the characteristics of the “prone stretching and adjusting neck manipulation”, the left-handed pulling trigger was performed first and then the right-handed pulling trigger was performed. During the manipulation process, the subjects were required to change positions once, and the time of position change varied among subjects, which affected the overall time of the manipulation, so the overall time of the manipulation was not counted. The time-force graph of Force Table 2 shows that there is a peak value of 514.1 N in the Z-axis direction at 5.68s, which occurs in all 10 subjects at the corresponding stage. Further analysis of the video recordings during the study showed that this phase was the process of the subject’s postural shift, in which the subject would raise his head and turn to the right side, and when he raised his head, the load on the force Table 1 would be significantly reduced and therefore a trough would appear in the Z-axis direction; when he raised his head, the pressure on the chest would increase, and the load on the force Table 2 would be significantly increased, and therefore a peak would appear in the Z-axis direction. Considering that the valleys and peaks at this phase are caused by the subject’s position change and are not related to the manoeuvre, they were not considered in the quantitative analysis of the manoeuvre.

## Limitations

In this experiment, 10 patients with cervical spondylotic radiculopathy were recruited as subjects, and there is a problem of insufficient sample volume, which may affect the accuracy of the quantitative parameters of the outcome indexes, and there is still a need to continue to carry out a large sample size study at a later stage. When the Motion Capture System is used, the Marker points are attached to the body surface, and the skin and muscles themselves are elastic, coupled with the contraction and stretching of the muscles caused by the manipulation of the manoeuvre, the position of the Marker points will have some displacement, which will lead to errors in the measurement. This may consequently affect the reported accuracy of certain kinetic and kinematic parameters. Although an experienced clinician was employed in this study, the inherent variability in manual manipulation techniques poses a significant barrier to clinical translation. As a preliminary exploratory study, its primary objective is to provide clinically standardised training for beginners in manual techniques, whilst offering a reference for safe and feasible ranges of motion during their implementation. Further research will be conducted to refine and expand upon these findings.

## Conclusion

This part of the experiment is based on a three-dimensional force measuring table system and motion capture technology to quantify the kinematic parameters of the “prone stretching and adjusting neck manipulation” manoeuvre, which is divided into the stretching phase, the trigger phase and the return phase, with a brief operational duration. The manoeuvre involved both the active hand and the assisting hand, and the vertical loading force of the active hand was consistent in different directions of the manoeuvre. The biomechanical effect of the manoeuvre on the cervical spine may help to reduce nerve root compression, thereby relieving symptoms in patients with cervical spondylotic radiculopathy.

## Data Availability

The original contributions presented in the study are included in the article/Supplementary Material, further inquiries can be directed to the corresponding author.
